# Governing transgender athletes in global sport: fairness, evidence, media representation, and lived experience

**DOI:** 10.3389/fspor.2026.1648181

**Published:** 2026-05-26

**Authors:** Yi Chen Chiu, Ya Chun Shen

**Affiliations:** Department of Leisure and Recreation Management, Taipei City University of Science and Technology, Taipei, Taiwan

**Keywords:** competitive sport, fairness, inclusion, lived experience, sport governance, transgender athletes

## Abstract

As gender diversity and gender identity have become increasingly central to contemporary public debate, the participation of transgender athletes in competitive sport has emerged as a highly contested issue at the intersection of science, law, governance, media, and human rights. Rather than treating this controversy as a simple opposition between fairness and inclusion, this article offers an integrative review of the major tensions shaping current debate. Drawing on academic studies, policy documents, media analyses, and first-person accounts published between 2015 and 2026, the article examines the issue through four interrelated dimensions: medical and physiological evidence, policy and legal developments, media and social narratives, and lived experience. The review shows that while testosterone, pubertal development, and related physiological factors remain relevant to certain aspects of athletic performance, the evidence remains limited, uneven, and highly variable across sports and performance indicators. It further demonstrates that sport policy is shaped not by science alone, but by the interaction of governance priorities, legal culture, political climate, and public discourse. In particular, the policy trajectory from the IOC's 2015 testosterone-based threshold model, to its 2021 principle-based framework, and further to its 2026 shift toward a more centralized and explicitly sex-based eligibility rule illustrates the instability of current governance approaches. The review also shows that mainstream and social media actively shape the public legitimacy of transgender athletes, while first-person accounts reveal embodied pressures, institutional exclusion, and forms of agency often overlooked in abstract policy debate. This article argues that transgender athletes’ participation should be understood not as a narrow eligibility issue, but as a complex governance problem involving knowledge translation, institutional design, procedural justice, and lived experience. A more sustainable policy direction requires interdisciplinary evidence, procedural transparency, sport-sensitive governance, and the meaningful inclusion of transgender athletes’ voices.

## 1 Introduction

The eligibility of transgender athletes to participate in competitive sport has emerged as a major issue at the intersection of contemporary sport governance, gender politics, and human rights debates. As gender identity and gender diversity increasingly move to the center of public policy and institutional design, traditional systems of sport classification based on binary biological sex have come under unprecedented scrutiny and challenge (1). However, controversies surrounding transgender athletes cannot be reduced to a single issue or understood as a straightforward clash between two opposing positions. Rather, they constitute a multi-layered problem involving sport physiology, institutional fairness, legal legitimacy, media representation, public affect, and the lived experiences of transgender individuals.

Existing discussions often emphasize either gender self-identification, dignity, and equal participation, or the question of fairness in competitive classification, particularly whether transgender women who have experienced male puberty may retain relative advantages in muscle mass, skeletal structure, strength output, and cardiorespiratory capacity (2–4). Yet framing this issue solely as a binary opposition between “human rights” and “fairness” risks oversimplifying the controversy and obscuring the multiple meanings of inclusion, safety, participation, and competitive integrity across different institutional contexts.

Accordingly, this article argues that the central challenge is not to elevate one value above all others, but to examine how tensions among competing values are co-constructed through scientific research, policy regulation, media representation, and public discourse. In this sense, transgender athletes' participation should be understood not simply as a question of eligibility, but as a complex governance issue shaped by institutional design, political climate, cultural anxiety, and embodied experience.

A significant policy shift occurred in March 2026, when the International Olympic Committee (IOC) introduced its Policy on the Protection of the Female (Women's) Category in Olympic Sport. Unlike the IOC's 2021 framework, which largely left eligibility decisions to individual international federations, the 2026 policy limits eligibility for the female category at the Olympic Games and other IOC events to biological females, with initial determination based on a one-time SRY gene screening. The IOC stated that this policy is intended to protect fairness, safety, and integrity in the female category, and that it will apply from the LA28 Olympic Games onward, without retroactive effect and without extending to grassroots or recreational sport. This development underscores the extent to which the governance framework surrounding transgender participation in sport remains dynamic and contested (5).

Despite substantial discussion from medical, regulatory, and media perspectives, at least three limitations remain in the existing literature. First, some studies rely too heavily on a single physiological indicator or a single policy text, resulting in an insufficient understanding of the multi-factorial nature of transgender participation in sport. Second, many accounts continue to frame the issue through binary opposition, without adequately examining the interaction among institutional fairness, human rights protections, and sport-specific differences. Third, compared with the extensive debate surrounding policy and media discourse, the first-person voices and lived experiences of transgender athletes often remain secondary in review-based scholarship.

In response to these gaps, this article adopts an integrative review approach to synthesize academic studies, policy documents, and representative public discourses on transgender athletes’ participation published between 2015 and 2026. The review is organized around four interrelated dimensions: medical and physiological evidence, policy and legal developments, media and social narratives, and lived experience. The purpose is not to endorse any single policy position, but to develop a more analytically robust framework for understanding how controversies over transgender athletes' participation are continuously reconstructed through the interaction of scientific uncertainty, governance demands, cultural imaginaries, and human rights claims. Through this integrative examination, the article seeks to provide a more nuanced reference point for future sport policy discussions and to suggest that sustainable institutional reform must be grounded in interdisciplinary evidence, sport-specific contexts, and the lived experiences of those most directly affected.

## 2 Medical and physiological dimensions: the controversy over testosterone levels and athletic performance

### 2.1 The role of testosterone in physiological difference

Testosterone, a sex hormone primarily produced by the testes, plays a central regulatory role in human physiology and has long been treated as a key explanatory factor in discussions of sex-based differences in athletic performance. It is closely associated with muscle mass, erythropoiesis, bone density, and athletic performance (6–11), and therefore constitutes an important physiological basis for the average performance advantages observed in males across certain domains of physical capacity. Importantly, these advantages do not arise from hormone levels measured at a single moment, but from the cumulative effects of long-term testosterone exposure on muscle, blood, skeletal structure, and overall body composition.

From the perspective of exercise physiology, the significance of testosterone lies in the concrete pathways through which it influences athletic capacity. Harper et al. (12) noted that testosterone promotes erythropoiesis, thereby increasing hemoglobin concentration and hematocrit, both of which are directly related to oxygen transport and endurance performance. Testosterone is also associated with increases in lean body mass, muscle cross-sectional area, and muscular strength, making it particularly relevant to strength- and speed-oriented events. In this sense, testosterone functions not simply as a biochemical marker, but as part of a broader physiological system through which performance-related capacities are developed and maintained.

At the same time, recognizing the importance of testosterone does not mean that it can independently account for all differences in athletic performance. Hamilton et al. (13) showed that while absolute differences between groups were often observed, most gender-related differences were attenuated after adjustment for body mass or other relevant anthropometric variables. Their analysis further suggested that transgender men generally performed more similarly to cisgender men, although typically as smaller men, and that transgender women likewise performed more similarly to cisgender women when body size and composition were taken into account. Accordingly, testosterone should not be treated as a stand-alone explanation for competitive advantage; rather, athletic performance is shaped by the interaction of multiple physiological and contextual factors, including body composition, hematological variables, pulmonary function, training history, and sport-specific demands.

A more analytically robust position, therefore, is neither to deny the relevance of testosterone nor to treat it as a self-sufficient explanation of competitive difference. Instead, it should be understood as an important but not exclusive physiological variable within a larger performance system.

### 2.2 Testosterone regulation policies for transgender athletes and their physiological premises

The governance of transgender women's participation in women's competitive sport has long been organized around testosterone regulation, with the underlying premise that testosterone is sufficiently linked to athletic performance to serve as a basis for eligibility classification. In this respect, testosterone policies have functioned not merely as medical guidelines, but as institutional mechanisms through which sport organizations translate physiological assumptions into rules of participation.

This rationale was clearly reflected in the IOC's 2015 policy, which required transgender women wishing to compete in women's events to maintain a total serum testosterone level below 10 nmol/L for at least 12 months prior to competition (14, 15). At the time, this threshold-based model appeared to offer an administratively workable solution by providing a measurable and ostensibly objective criterion for eligibility. Yet the apparent clarity of such a standard also depended on a broader institutional assumption: namely, that fairness in women's sport could be adequately protected through the regulation of a single biomarker. It is precisely this assumption that has become increasingly contested.

Following the release of the Framework on Fairness, Inclusion and Non-Discrimination on the Basis of Gender Identity and Sex Variations in 2021, the IOC moved away from endorsing a single testosterone threshold as a universal rule applicable across all sports. Instead, it adopted a principle-based framework centered on fairness, inclusion, and non-discrimination, while delegating the formulation of specific eligibility criteria to individual international federations (16, 17). This shift is analytically significant because it suggests recognition that no single physiological threshold can easily resolve the diverse competitive demands of different sporting contexts.

More recent policy developments indicate that the institutional authority of testosterone-based regulation has weakened further. While the 2021 IOC framework moved away from a universal hormonal threshold, the IOC's 2026 policy shift signaled a broader move toward sex-based eligibility rules for the female category at IOC events. At the same time, policy developments in other contexts, including the United States, have shown that eligibility rules are increasingly shaped not only by physiological reasoning but also by political restriction and legal intervention. Taken together, these developments suggest that the current global landscape is defined less by regulatory convergence than by continuing tension among hormonal regulation, sex-based classification, and competing institutional conceptions of fairness.

Testosterone regulation has therefore remained central to the governance of transgender athletes, but it can no longer be treated as a settled or sufficient solution. The key issue is no longer simply whether testosterone has physiological relevance, but whether any single biological indicator can be translated into a stable, universally legitimate, and sport-governing rule of classification.

### 2.3 Points of contention and critique

The controversy surrounding transgender women's participation in women's competition primarily centers on two related, but distinct, questions. The first concerns whether transgender women who have experienced male puberty retain physiological advantages sufficient to affect competitive outcomes after undergoing GAHT. The second concerns whether policy frameworks centered on testosterone levels can provide an adequate basis for fair classification.

With regard to the first question, recent studies provide stronger evidence than was available in earlier discussions. Roberts et al. (4) found that, following feminizing hormone therapy, transgender women gradually showed declines in push-up and sit-up performance that approached the levels of cisgender women, whereas a relative advantage in 1.5-mile running performance remained evident after two years. Chiccarelli et al. (18) similarly reported that different fitness indicators do not change at the same rate after GAHT. In addition, Harper et al. (12) showed that hemoglobin and hematocrit typically decline to the cisgender female range within three to four months, while lean body mass, muscle cross-sectional area, and strength often remain higher than those of cisgender women even after 36 months. Harper et al. (19), using longitudinal data on transgender women runners and swimmers, likewise found that athletic performance declined after GAHT, with greater reductions generally observed in longer-duration endurance events than in shorter events. Taken together, these findings suggest that GAHT affects endurance-related and strength-related systems through different time courses, and that retained or reduced advantages may vary substantially across sporting events.

With regard to the second question, however, the current evidence remains insufficient to support any overly simplified institutional conclusion. Many studies are based on limited sample sizes, most do not focus on elite athletes, and some rely on cross-sectional designs, retrospective data, or self-reported training volume. Although there is now relatively consistent directional evidence that testosterone and GAHT affect muscle mass, hematological markers, and some aspects of physical performance, these findings do not justify generalized conclusions across different sports, competitive levels, and regulatory contexts. In other words, the current literature is sufficient to reject the claim that testosterone is irrelevant, but it remains insufficient to support the assumption that a single testosterone threshold can resolve all questions of fairness.

Accordingly, testosterone should neither be minimized as an inconsequential numerical marker nor institutionalized as the sole proxy for fairness across all sporting events. The real issue is not whether testosterone matters, but how limited and sport-variable physiological evidence can be translated into participation policies that are legitimate, operationally workable, and proportionate. This is one of the key reasons why international sport governance has thus far struggled to produce a stable consensus on this issue.

## 3 Policy and legal dimensions: regulatory developments in international sport organizations

### 3.1 The policy trajectory of the international Olympic committee

The evolution of the IOC's policies on transgender athletes reflects the broader ways in which international sport governance has adjusted its institutional logic in response to tensions among gender diversity, human rights protection, and competitive fairness. Broadly speaking, this evolution can be divided into four stages. The first was an access model grounded in medical and legal recognition. The second shifted toward eligibility management centered on physiological indicators. The third developed into a governance framework emphasizing general principles and sport-specific differentiation. A fourth and more recent stage has signaled renewed central coordination and a more explicitly sex-based eligibility approach for the female category.

The 2003 Stockholm Consensus required transgender athletes to undergo sex reassignment surgery, complete at least two years of hormone treatment, and provide legal recognition of their affirmed sex before being permitted to compete in accordance with their gender identity (20). The defining feature of this model was its attempt to establish clear boundaries of participation through medical intervention and legal documentation, thereby responding to the sport system's demand at the time for stable sex classification. However, this model soon attracted criticism because it relied too heavily on surgery and legal recognition. It was seen as excessively intrusive in relation to bodily autonomy and difficult to implement consistently across countries with differing legal systems, medical resources, and regulatory conditions (20).

Against this backdrop, the IOC revised its policy in 2015 by removing the surgical requirement and replacing it with testosterone concentration as the primary criterion for eligibility. Transgender women seeking to compete in women's events were required to maintain testosterone below a specified threshold for a prescribed period. The institutional significance of this shift lay in the fact that the IOC no longer treated the completion of sex transition as the central standard, but instead adopted a physiological metric as a more operational basis for eligibility. This change reduced the medical burden imposed under the earlier model and provided many international sport federations with a regulatory template that could be more readily adopted. Similar approaches were subsequently reflected in the policies of organizations such as World Athletics and FINA (21, 22). Yet the 2015 model was also quickly criticized, because a single testosterone threshold could not adequately account for differences across sporting events, variation in individual responses to hormone treatment, or the fact that the relationship between testosterone and actual athletic performance is not linear (17).

It was in response to dissatisfaction with such uniform thresholds that the IOC released the *Framework on Fairness, Inclusion and Non-Discrimination on the Basis of Gender Identity and Sex Variations* in 2021. Rather than prescribing a universal testosterone threshold or surgical requirement, the IOC replaced a uniform rule with a principle-based framework and argued that individual sport federations should formulate eligibility criteria according to the characteristics of their sport, the level of risk involved, and the evidence available (17). From the perspective of institutional design, this marked a move toward differentiated governance by sport. However, the limitation of the 2021 framework lay not in its principles as such, but in the fact that it delegated rule-making authority to individual sport bodies without simultaneously providing sufficiently clear minimum procedural requirements, standards of evidence, or proportionality principles. As a result, substantial divergence in implementation became possible, with some organizations interpreting the framework as creating space for sport-specific governance and ongoing monitoring, while others adopted more explicit forms of precautionary exclusion and stricter eligibility classifications (23–25).

This interpretation is further supported by the IOC's subsequent actions. In June 2025, the newly elected IOC President Kirsty Coventry stated that the IOC would once again take a more active coordinating role in relation to sex eligibility standards, rather than leaving responsibility entirely to individual international federations (26). In September 2025, the IOC established a Working Group on the Protection of the Female Category to address controversies concerning transgender participation and the protection of women's categories (27). That process culminated in a formal policy shift on 26 March 2026, when the IOC introduced its *Policy on the Protection of the Female (Women's) Category in Olympic Sport* (5). Under this policy, eligibility for the female category at the Olympic Games and other IOC events, including the Youth Olympic Games and IOC qualification events is restricted to biological females. Initial eligibility is determined through a one-time SRY gene screening intended to identify male sex development. The IOC stated that the policy was adopted to protect fairness, safety, and integrity in the female category, that it will take effect from the LA28 Olympic Games onward, and that it is neither retroactive nor applicable to grassroots or recreational sport. In effect, the 2026 policy marks a clear departure from the decentralized logic of the 2021 framework, as the IOC moved from delegating eligibility rule-making to international federations toward reasserting a centralized, sex-based standard for the female category across IOC events.

### 3.2 From flexible frameworks to restrictive policies: the policy shift of world athletics and the NCAA

At the level of international sport federations, World Athletics has, since 2023, explicitly prohibited transgender women who have undergone male puberty from competing in women's international events (25). Its regulatory logic no longer rests solely on the management of testosterone levels, but instead advances the position that certain physical characteristics shaped by male puberty cannot be fully eliminated through subsequent hormone suppression. On this view, eligibility determinations based only on testosterone thresholds remain insufficient to preserve competitive comparability within the women's category (25, 28). Compared with the IOC's 2021 framework, which emphasized human rights principles, non-discrimination language, and sport-specific governance, the approach adopted by World Athletics reflects a more explicit logic of categorical clarity and precautionary exclusion, indicating that regulatory certainty and competitive fairness have been placed in a more prioritized position (17, 28). This contrast has become less sharp since the IOC's own 2026 shift toward a more centralized and explicitly sex-based eligibility model, suggesting that sport-specific governance has not necessarily led to more inclusive outcomes in practice.

By contrast, the policy development of the NCAA more clearly demonstrates that sport regulation is shaped not only by governance considerations internal to sport, but also by the broader domestic political and educational policy environment. In 2022, the NCAA adopted what it described as a sport-by-sport approach, which formally echoed the IOC's encouragement of differentiated governance by allowing different sports to adjust transgender eligibility requirements according to the standards of their respective international federations (29). Yet this differentiated model did not signal a departure from physiological verification as a regulatory logic. On the contrary, the policy continued to require transgender women to provide documentation of testosterone suppression and to undergo ongoing monitoring before and during the competitive season (23). Accordingly, the claimed flexibility of this model reflected more an adjustment in regulatory implementation than a fully inclusive shift in normative orientation (23, 24).

This regulatory direction became more visibly restrictive in 2025. On February 6, 2025, the NCAA Board of Governors announced a new participation policy stating that women's competition would be limited to student athletes who were assigned female at birth. Those assigned male at birth would still be permitted to practice with women's teams and receive related benefits, but would not be allowed to participate in official women's competition (30).

More importantly, the NCAA revision must be situated within the broader context of rapidly tightening U.S. federal policy in early 2025 (30, 31). On January 20, 2025, the Trump administration issued Executive Order 14168, *Defending Women From Gender Ideology Extremism and Restoring Biological Truth to the Federal Government*, which required federal agencies to treat binary and immutable biological sex as the basis for administration and policymaking (31). This was followed by Executive Order 14201, *Keeping Men Out of Women's Sports*, which extended the same logic into the domains of education and women's sport and called for the withdrawal of federal funding from educational programs that were deemed to deprive women and girls of fair athletic opportunities (32). The NCAA's immediate policy revision after this order indicates that the tightening of eligibility rules should be understood not merely as a sport-specific regulatory adjustment, but as part of a broader political and legal intervention into the governance of athletic participation.

From a comparative perspective, although World Athletics and the NCAA operate at different governance levels, with the former being an international sport federation and the latter a governing body for collegiate sport in the United States, both ultimately moved toward treating biological sex or pubertal experience as the primary logic for eligibility in the women's category (25, 30). This suggests that the flexible framework opened by the IOC in 2021 did not produce a stable trajectory toward inclusive regulation in subsequent practice. Rather, in highly contested settings, it was increasingly translated into more explicit forms of exclusionary classification. This trend is reinforced by the IOC's own 2026 policy shift. Put differently, the IOC's 2021 framework provided a principled basis for regulatory transition, but it did not establish a durable institutional trajectory toward inclusion; instead, subsequent developments across both international and domestic sport governance increasingly favored clearer, more restrictive, and more biologically anchored category boundaries.

### 3.3 National policy examples and human rights challenges

When the analytical lens is extended from international sport organizations to the national level, it becomes even clearer that controversies over transgender athletes' participation are not determined solely by sport science or by the rules of a single governing body. Rather, they are shaped simultaneously by legal culture, political structure, and prevailing conceptions of human rights (17). Differences in national policy therefore reflect more than technical variation in regulation. They also reveal how different systems understand the relationship among equality, protection, procedural justice, and gender justice.

The United States provides a particularly clear example. For many years, transgender athlete policy in the United States was shaped by the interaction between federal structures and state-level variation. By early 2025, however, developments at the federal level showed that the issue had expanded beyond a dispute over sport regulation and had become part of a broader restructuring of gender governance and executive authority. Executive Order 14168 first required federal agencies to treat binary biological sex classification as the basis for policy and administrative documentation (31). Executive Order 14201 then extended this logic into the fields of women's sport and educational funding, with Title IX and federal funding serving as key regulatory instruments (32). This suggests that the current tightening of policy in the United States is not merely organized around competitive fairness, but is also closely tied to the federal government's attempt to redefine sex, the protection of women, and the boundaries of institutional recognition (31, 32). For this reason, transgender athletes' eligibility in the United States is no longer only a matter of sport governance, but has become a symbolic issue within the broader dynamics of culture war and partisan politics (30).

By contrast, policy discussions in the United Kingdom have more often been framed in terms of risk management and sport-specific differentiation. These discussions emphasize designing rules according to the degree of contact, the level of injury risk, and the competitive characteristics of particular sports, thereby reflecting a stronger orientation toward procedural rationality and graded risk assessment (33). Yet although this institutional language does not tighten restrictions through executive order in the same direct and rapid manner seen in the United States, actual implementation may still leave transgender athletes in a prolonged condition of uncertainty because standards vary across sport organizations. This illustrates that flexibility does not automatically guarantee inclusion. In the absence of clear procedural requirements, appeal mechanisms, and evidentiary standards, differentiated governance may equally produce instability and opacity.

New Zealand, by comparison, has demonstrated a more explicit rights-based orientation at the level of community sport. In 2022, Sport NZ issued its *Guiding Principles for the Inclusion of Transgender People in Community Sport*, emphasizing that transgender people should be able to participate in community sport according to their self-identified gender without first having to satisfy stringent medical verification requirements (34). This policy direction more fully embodies the principle of inclusion in recreational and community sport and offers an alternative governance imagination distinct from that of high-performance sport. However, once participation moves into elite sport, regulatory frameworks still need to align with the standards of international sport federations, and greater restrictions therefore remain in place in highly competitive contexts. This demonstrates that even when a country adopts an inclusive value orientation at the policy level, a substantial institutional gap may still persist between community sport and elite sport.

Taken together, these national examples show that policy differences reflect distinct ways in which institutional systems define risk, evidence, and justice. The key question at the legal and policy level is therefore not only whether a given category of athlete should be allowed or prohibited from competing, but also who is included in decision-making processes, which forms of evidence are treated as valid, which risks are amplified, and which lived experiences are excluded from institutional consideration. In this respect, the limitation of the IOC's 2021 framework lay not only in its failure to provide a uniform answer, but also in the fact that, after devolving authority, it did not establish a sufficiently clear framework for procedural justice, evidentiary standards, and institutional accountability. The IOC's subsequent 2026 shift toward a more centralized and sex-based eligibility model can be understood, in part, as a response to these unresolved problems, even though it has also generated new concerns regarding privacy, inclusion, and the governance of biological verification. A more legitimate direction for future governance would therefore lie neither in relying exclusively on a single universal threshold nor in remaining at the level of abstract inclusivity. Instead, it would require a more stable, transparent, and reviewable institutional design grounded in interdisciplinary evidence, procedural safeguards, and the meaningful participation of those most directly affected.

## 4 Social and media narratives: the construction of the public image of transgender athletes

### 4.1 How mainstream media represent transgender athletes

The representation of transgender athletes in mainstream media is not merely a matter of reporting individual events. It also shapes how the public understands the legitimacy of their participation, their gender identity, and their institutional position in sport. Existing research shows that mainstream media coverage often revolves around several recurring frames, including heroization, controversy, and problematization. Although these frames may appear distinct, they all contribute to the construction of transgender athletes' public image and influence how society defines their acceptability and legitimacy (35–37).

Heroizing narratives often portray transgender athletes as exemplary figures who overcome adversity and persist in pursuing their aspirations. Fallon Fox, for example, has at times been represented as a courageous figure who disclosed her identity, challenged male-dominated sport spaces, and spoke on behalf of the transgender community (35). Yet such narratives may also reduce institutional exclusion and cultural discrimination to a story of individual perseverance, thereby obscuring the structural conditions under which transgender athletes are repeatedly required to prove their legitimacy. In this sense, heroization does not necessarily signal genuine acceptance; it may instead rest on the expectation that the athlete must continue to endure scrutiny and self-justification.

A more common mode of coverage places transgender athletes within frames of controversy and risk. Relevant studies indicate that news texts frequently focus on questions such as whether transgender athletes possess an unfair advantage, whether they threaten women's sport, and whether existing rules contain regulatory loopholes. Through headline construction, quotation selection, and emotionally charged language, transgender athletes are often positioned as focal points of institutional crisis (38, 39). Even when exclusion is not stated explicitly, interrogative framing, unattributed quotations, and biologically essentialist language may normalize doubt and reinforce public anxiety toward transgender participation in sport.

This narrative pattern is especially evident in the case of Laurel Hubbard. Kavoura and Jenzen (40), in their analysis of British mainstream media coverage of Hubbard's participation in the Tokyo Olympics, found that the media simultaneously portrayed her as a competitive threat associated with residual male advantage and questioned the legitimacy of the rules themselves, even while acknowledging that she complied with the eligibility regulations in force at the time. As a result, Hubbard was not merely reported as an athlete, but was constructed as a symbolic challenge to sport institutions, women's safety, and scientific rationality. Notably, transgender voices were often relatively absent from this kind of coverage, suggesting that mainstream media more often positioned transgender athletes as vehicles of controversy than as subjects with narrative agency.

Taken together, mainstream media do not simply reflect pre-existing attitudes toward transgender athletes. Rather, they actively shape public image and institutional legitimacy through recurring narrative frames. Heroization, controversy, and problematization may differ in tone, but all can keep transgender athletes in a position of being watched, interpreted, and scrutinized.

### 4.2 Social media platforms and public perception

If mainstream media tend to organize the public image of transgender athletes through established news frames, social media platforms further amplify, circulate, and reconfigure these controversies. The openness and high interactivity of platforms such as Twitter, YouTube, Facebook, and Reddit mean that debates over transgender athletes are no longer shaped solely by news organizations. Instead, they become discursive arenas in which large numbers of users, opinion leaders, and advocacy groups participate simultaneously. Within these spaces, supportive and opposing positions often gather rapidly and confront one another directly, making terms such as fairness, inclusion, protection of women, and human rights more likely to be used in emotionalized, labeled, and polarized ways. Relevant studies suggest that such discussions tend to generate confrontational narratives and mobilized positions (41–43).

Research further indicates that these discussions often develop along two competing discursive directions. On the one hand, some users emphasize that participation according to gender identity reflects respect for diverse identities and basic rights. On the other hand, other narratives argue that women's sport must be protected and ground their claims in physiological difference, competitive comparability, and the maintenance of institutional boundaries. Murib's (43) analysis of YouTube comments showed that many users framed their skepticism toward transgender athletes as neutral, scientific, or common-sense judgment, thereby legitimizing exclusionary positions in the name of biological fact. Avalos et al. (41) similarly found that responses to transgender Olympians on social media were marked by considerable negative perception and hostile discourse, while supportive voices remained comparatively limited. Social media platforms therefore do not merely place different viewpoints side by side; they also amplify some positions more effectively through emotion, visibility, and repetition.

At the same time, social media platforms carry a dual significance for transgender individuals themselves. Research shows that platforms such as YouTube can provide spaces of emotional support, identity affirmation, and community connection for LGBTQ users. Engagement with LGBTQ creator content has been positively associated with social connectedness, self-esteem, and collective self-esteem, suggesting that digital platforms can play a constructive role in identity formation and emotional support (44). However, this does not mean that such platforms are inherently protective. When the issue of transgender athletes enters highly contentious discursive contexts, social media may also become a source of hate speech, negative comparison, renewed stigma, and emotional strain, thereby exposing more vulnerable or not yet openly identified individuals to additional risks (43, 44).

More broadly, the interactional logic of platforms can intensify the circulation of controversy itself. Calabrese et al. (42) found that, on Twitter, negative affect was clearly associated with higher post engagement in discussions related to transgender athletes, indicating that emotions such as anger, anxiety, and hostility may function not only as expressions of opinion but also as mechanisms for increasing visibility and dissemination. Social media therefore do not simply reflect existing attitudes toward transgender athletes. Through the combined operation of algorithms, interactivity, and emotional mobilization, they also shape how the issue is publicly understood, making it more likely to be framed as a conflict that demands taking sides rather than as an institutional and experiential issue requiring careful discussion (42, 43).

### 4.3 The interaction between media representation and social acceptance

Media coverage of transgender athletes does not merely reflect pre-existing social positions. It also influences which issues come to be seen as important, which emotions are regarded as legitimate, and which subjects are positioned as objects of suspicion. When mainstream media repeatedly associate transgender athletes with a fairness crisis, threats to women's sport, or regulatory loopholes, these narratives do more than shape public perception; they may also provide cultural legitimacy for more restrictive policy directions (38, 40).

This process is not unidirectional. Media reporting often responds to existing political climates and social anxieties, but may also amplify and stabilize them. When news coverage and online discussion consistently revolve around the protection of women's sport, while giving comparatively little attention to the actual participation experiences, institutional pressures, and everyday lives of transgender athletes, the public is more likely to understand transgender people as the source of institutional problems rather than as stakeholders within contested institutional arrangements (35, 38).

Social media platforms further accelerate this cycle. Research indicates that the interactional dynamics of platforms, together with the circulation advantage of high-conflict content, make issues related to transgender athletes more likely to be expressed in polarized ways and to generate highly oppositional public emotions in a short period of time (44, 45). Under these conditions, media narratives, public affect, and policy pressure often become mutually reinforcing: media amplify controversy, social anxiety intensifies, and policymakers become more likely to adopt regulatory approaches that provide clearer classificatory boundaries but also produce stronger exclusionary effects.

Accordingly, media cannot be treated simply as external observers, nor can analysis remain limited to individual news texts. Media representation and platform circulation must instead be understood as part of the broader apparatus of institutional governance. In this context, media do not merely transmit information; they actively shape the boundaries of acceptability, legitimacy, and exclusion. Social acceptance, in turn, is continuously constructed and adjusted through these ongoing processes of representation, response, and amplification.

## 5 Lived experience: subjectivity and challenges of transgender athletes

### 5.1 Subjective motivations for sport participation, identity affirmation, and first person experience

For transgender athletes, participation in sport is not only related to health, challenge, self-realization, and a sense of group belonging, but also to whether their identity can be recognized and enacted within a given setting. Sport is therefore not merely a space for physical activity or competitive performance; it is also a social arena in which transgender individuals continually negotiate whether they can exist in ways consistent with their affirmed identity. Whereas many other athletes may experience participation primarily as an extension of interest or an opportunity to demonstrate ability, transgender individuals often confront an additional and more sensitive question: whether they can enter facilities, use changing spaces, train, compete, and be treated by others in ways that align with their gender identity.

For this reason, first-person studies repeatedly show that the central issue for transgender individuals in sport is not simply whether they are allowed to participate, but whether they are recognized as legitimate and natural participants. Being addressed correctly, wearing equipment consistent with one's gender identity, or using relevant spaces without disruption are not minor details; they are critical moments through which one is recognized as belonging in that environment (46). This suggests that sport participation is not only a matter of formal access, but also one of visibility, recognizability, and legitimacy.

More importantly, such identity affirmation is not achieved once and for all. It unfolds across life stages, spatial settings, and interpersonal interactions. Qualitative studies indicate that transgender individuals' experiences in sport and physical activity are often deeply intertwined with different moments of transition, from the period before coming out, to the disclosure of identity, to the gradual formation of a more stable sense of self (47). Sport is therefore often not simply a question of whether one can join, but of how visibility, position, and safety are negotiated over time.

First-person experience in sport should thus not be reduced to a binary of recognition vs. exclusion. It is better understood as a continuous process of identity negotiation. Narrative studies show that the stories of transgender and gender nonconforming athletes often revolve around gender sanction, survival within binary institutions, disclosure and transition, and moments of affirmation (48). Sport is not merely the setting in which an already established gender identity is displayed; it is also one of the settings in which that identity is gradually articulated, felt, and enacted.

In addition, first-person research reminds us that transgender athletes' sporting experiences are deeply temporal and embodied. Whether in collegiate competition, where an athlete may move from a women's team to a men's team, or in weight training and fitness spaces, where equipment, movement, body contour, and the gaze of others repeatedly shape one's sense of position, belonging is rarely a stable status that is simply acquired. Rather, it is continuously constructed through emotional fluctuation, self-doubt, social support, and spatial interaction (49, 50).

Taken together, qualitative research suggests that transgender participation in sport cannot be adequately understood through policy texts or physiological data alone. For transgender athletes, participation is itself a continuous process of negotiating institutional boundaries, social imaginaries, and embodied realities. The significance of first-person experience lies precisely in its ability to show that transgender athletes are not abstract cases in public debate or passive objects of governance, but individuals who bear the consequences of institutional arrangements and respond to them through their bodies and actions.

### 5.2 Barriers and exclusion across the competitive journey

The difficulties faced by transgender athletes throughout their competitive journey are rarely confined to a single event or isolated conflict. More often, they are produced through the combined effects of institutional regulations, gendered culture, spatial arrangements, and everyday interactions. From the perspective of lived experience, the central barrier is not simply whether participation is formally prohibited, but whether transgender athletes can exist within sporting spaces as ordinary participants without being repeatedly questioned, exposed, or marginalized.

Existing review studies show that negative experiences in sport among transgender individuals are not sporadic, but structurally recurring. Reviews and qualitative meta-syntheses indicate that transgender people repeatedly encounter non-inclusive environments, gendered language, feelings of unsafety in facilities and spaces, and experiences of rejection arising from the mismatch between their bodies or identities and dominant gender expectations (51, 52). Changing rooms, restrooms, swimming pools, gyms, and team spaces are particularly significant in this regard, because they are not merely ancillary facilities of sport, but everyday sites where gender order is actively reproduced. Much of the pressure transgender athletes experience does not arise from explicit prohibition, but from persistent uncertainty about whether they will be noticed, questioned, misgendered, outed, or treated as someone who does not belong.

As a result, the position of transgender athletes within the competitive process often takes the form of continuous self-management. Qualitative studies show that many transgender and gender nonconforming athletes rely on concealment, stealth, passing, or the suppression of gender expression in order to remain involved in highly binary sport systems (47, 48). This suggests that being able to stay does not necessarily mean being genuinely accepted; it may instead reflect a survival strategy for temporarily avoiding conflict. Exclusion in sporting spaces therefore does not always take the form of formal rules or overt hostility. It may also be enacted more subtly through everyday interaction, gaze, humor, silence, equipment classification, and spatial organization.

This experience of exclusion is especially visible at the embodied level. First-person narratives and autoethnographic studies show that transgender athletes often have to monitor their body contour, clothing choices, movement style, and spatial position in order to reduce the risk of being read, questioned, or exposed (49). These accounts remind us that the pressure experienced during athletic participation does not arise only from policy thresholds or eligibility review, but also from how the body is present, how it is seen, and how it is inserted into existing systems of gender classification. When a seemingly technical choice—such as which changing room to enter, which equipment to use, where to warm up, or with whom to train—becomes a test of gender legitimacy, participation in sport itself becomes a form of social and psychological labor.

In addition, the competitive experience of transgender athletes is deeply shaped by team culture, coaching attitudes, and the accessibility of organizational resources. Case studies indicate that even when formal participation is permitted, transgender athletes may still experience significant emotional fluctuation, isolation, and uncertainty about the future during periods of transition if teammate support is limited, coaching responses are indifferent, or organizational resources are insufficient (50). The barriers they face should therefore not be reduced to a simple policy dispute, but understood as the outcome of interaction among institutional design, spatial order, cultural imaginaries, and interpersonal support.

Taken together, what transgender athletes confront throughout the competitive journey is not merely a question of eligibility, but a condition of ongoing institutional vulnerability. Beyond the demands of athletic performance itself, they must also bear additional burdens related to bodily management, risks of visibility, gender legitimacy, and spatial safety. Unless these burdens are understood from the perspective of lived experience, institutional discussions of fairness and inclusion are likely to remain at the level of abstract principle and fail to engage with the actual forms of exclusion that occur within competitive sport.

### 5.3 Voice, subjective experience, and advocacy

If the preceding sections show how transgender athletes bear institutional and cultural pressures, an equally important dimension concerns how they speak through their own experiences and gradually move from being regulated subjects to becoming participants in institutional debate.

The narratives of transgender athletes themselves have reflective and counter-narrative significance. Relevant studies show that these stories do not merely reveal gender sanction, strategies of survival within binary institutions, and risks associated with visibility. They also describe moments of being correctly addressed, receiving support from teammates, or regaining a sense of alignment between body and self through sport (48). Their importance lies in allowing transgender individuals to move beyond being defined solely by external experts, media institutions, or regulatory systems, and instead to explain in their own terms what exclusion, safety, and meaningful recognition actually look like. In this sense, subjective experience is not simply a record of suffering, but may also constitute an everyday challenge to existing gender order.

This agency is not limited to formal advocacy or public action. It is also present in more micro-level forms of everyday negotiation. Qualitative studies indicate that the ongoing adjustments transgender individuals make in language use, access to space, bodily presentation, and interactional strategy carry a certain resistant quality. On the one hand, these practices reflect how institutional pressure operates. On the other hand, they show how individuals maintain participation, rebuild position, and preserve subjectivity under constrained conditions (52). Advocacy, therefore, should not be understood only as public speaking or policy lobbying; it also includes the continuing negotiation of naming rights, existence, and the right to use space in everyday life.

More fundamentally, first-person writing and autoethnographic research remind us that experience itself can become a mode of knowledge production. When transgender athletes bring experiences such as membership, gaze, passability, belonging, and bodily anxiety into sport research through journaling, reflective writing, and embodied narrative, they are not only describing their own situations. They are also redefining how inclusion, space, and participation are to be understood (49). This means that transgender voices are no longer simply opinions to be consulted after institutional debates have already taken shape; they can become part of institutional analysis itself.

At a more public level, transgender athletes have also increasingly intervened in institutional reform and public discourse through media interviews, social media expression, collaboration with advocacy organizations, and participation in policy consultation. The significance of these actions lies not only in the expression of position, but in redirecting controversies often framed by external observers back toward institutional consequences and everyday experience. When transgender athletes explain how regulations affect their training, belonging, sense of safety, and future imagination, the focus of public discussion may shift from abstract classification and singular indicators toward questions of lived experience, procedural legitimacy, and institutional responsiveness.

Accordingly, if sport institutions are to enhance their legitimacy and social acceptability, they cannot continue to treat transgender athletes merely as subjects to whom rules are applied. They must more actively recognize them as providers of experiential knowledge and participants in institutional design. This does not mean that first-person experience can automatically resolve all tensions between fairness and inclusion. It does mean that, without the perspective of those most directly affected, institutional design is far more likely to overlook how regulations are actually felt, enacted, and borne in everyday sporting life. Including transgender athletes in institutional dialogue is therefore not simply a symbolic gesture of inclusion, but a necessary condition for improving policy responsiveness and procedural legitimacy.

## 6 Conclusion

This article has examined the controversies surrounding transgender athletes' participation through four interrelated dimensions: medical and physiological evidence, policy and legal developments, media and social narratives, and lived experience. Overall, the review argues that the issue cannot be resolved by any single physiological indicator or reduced to a binary opposition between human rights and fairness. Rather, it is a complex governance problem shaped by scientific uncertainty, institutional design, political context, cultural representation, and embodied experience.

At the medical and physiological level, existing evidence suggests that testosterone, male pubertal development, oxygen-carrying capacity, muscle mass, and strength are relevant to some aspects of performance, but findings remain inconsistent across sports, indicators, and treatment durations. At the policy and legal level, the governance of transgender athletes has never been driven by science alone. It has been shaped by the interaction of international regulatory logics, domestic politics, legal cultures, and institutional perceptions of risk. From the IOC's 2015 testosterone-based threshold model, to its 2021 principle-based and sport-specific framework, and further to its 2026 shift toward a more centralized and explicitly sex-based eligibility rule for the female category at IOC events, it is clear that sport governance has remained dynamic rather than settled.

At the media and social level, transgender athletes are continually constructed through narratives of heroization, controversy, and problematization, while social media intensifies polarization and compresses complex issues into simplified political labels. At the level of lived experience, transgender athletes are not merely objects of regulation, but subjects who directly bear the consequences of institutional arrangements. Any governance model that ignores these embodied realities risks becoming formally coherent but socially inadequate.

In sum, controversies over transgender athletes' participation should be understood as a multi-layered issue produced through the interaction of science, law, governance, media, and lived experience. A more sustainable direction for future governance should neither rely exclusively on a single physiological threshold nor remain at the level of abstract inclusion. Instead, it should seek a more transparent, reviewable, and context-sensitive framework grounded in interdisciplinary evidence, procedural safeguards, proportionality, and meaningful attention to those most directly affected. Future research should continue to examine physiological evidence across sports, regulatory change, and the diverse lived experiences of transgender athletes in order to better understand how institutional legitimacy is continually renegotiated.
